# Surveillance Recommendations in Reducing Risk of and Optimally Managing Breast Cancer-Related Lymphedema

**DOI:** 10.3390/jpm4030424

**Published:** 2014-08-18

**Authors:** Pamela L. Ostby, Jane M. Armer, Paul S. Dale, Margaret J. Van Loo, Cassie L. Wilbanks, Bob R. Stewart

**Affiliations:** 1 Sinclair School of Nursing, University of Missouri-Columbia, S235 School of Nursing Building, Columbia, MO 65211, USA; E-Mails: plo7c9@mail.missouri.edu (P.L.O.); stewartb@missouri.edu (B.R.S.); 2 Lymphedema Research Laboratory, Sinclair School of Nursing, University of Missouri, DC 116.05, Suite 408, Mizzou North Campus, Columbia, MO 65211, USA; 3 Ellis Fischel Cancer Center, One Hospital Drive, Columbia, MO 65212, USA; E-Mails: daleps@health.missouri.edu (P.S.D.); vanloomj@health.missouri.edu (M.J.V.L.); wilbanksc@health.missouri.edu (C.L.W.)

**Keywords:** breast cancer-related lymphedema, surveillance, risk-reduction, self-management

## Abstract

Breast cancer survivors are at increased risk for the development of breast cancer-related lymphedema (BCRL), a chronic, debilitating, and disfiguring condition that is progressive and requires lifelong self-management of symptoms. It has been reported that over 40% of the 2.5 million breast cancer survivors in the United States may meet the criteria for BCRL during their lifetimes. Ongoing surveillance, beginning with pre-operative assessment, has been effective in identifying subclinical lymphedema (LE). A prospective model for surveillance is necessary in order to detect BCRL at an early stage when there is the best chance to reduce risk or slow progression. Physical methods for monitoring and assessment, such as circumferential arm measures, perometry, bioimpedance; exercise programs; prophylactic and early-intervention compression garments; and referral for complete decongestive therapy are all interventions to consider in the development of a BCRL surveillance program. In addition, supportive-educative programs and interactive engagement for symptom self-management should also be implemented. The importance of interdisciplinary collaboration is integral to the success of an effective personalized medicine program in breast cancer-related lymphedema surveillance.

## 1. Introduction

### 1.1. Definition

Lymphedema is a chronic, debilitating condition with a variety of causes that can be diagnosed as a primary congenital condition, or it can be secondary from trauma to lymphatic structures. Secondary lymphedema can occur from scarring associated with wound healing, cancers causing tumor compression on lymphatic channels, surgical trauma to the lymphatic channels, fibrosis or scarring of the channels caused by radiation therapy, and infection [1,2,3,4,5,6]. Trauma to the lymphatic channels inhibits the ability for the lymphatic fluid to travel back to the blood circulatory system resulting in protein-rich lymphatic fluid accumulation in the extracellular spaces and swelling of the soft tissues [3,4,6]. The most common cause of secondary lymphedema in the United States is breast cancer treatment, including sentinel lymph node biopsy (SLNB), axillary lymph node dissection (ALND), mastectomy, or radiation therapy (RT) for treatment of breast cancer [5,6,7,8,9,10]. However, breast cancer-related lymphedema (BCRL) symptoms have been reported with all treatment modalities, including the absence of either ALND or SLNB [4]. 

### 1.2. Incidence

Although the emergence, incidence, and prevalence of BCRL are not fully understood, every breast cancer survivor is at risk [6,11]. Because there is no single standardized measure or universal criteria used to diagnose BCRL, reports vary with regard to BCRL incidence [12,13]. In addition, BCRL can occur within days or up to decades postoperatively in breast cancer survivors, which makes follow-up difficult in cases of delayed development [1,14]. In one cohort of 263 women followed over a 20-year period, Petrek* et al.* reported that 77% of cases studied who developed lymphedema did so within three years after surgery [15]; however, prevalence has been correlated to length of follow-up after breast cancer treatment [16].

It is estimated that over 40% of breast cancer survivors may meet the criteria for BCRL over the course of their lives [7,17]. In a study of 627 patients diagnosed with breast cancer who underwent 664 mastectomies between 2005–2013, a two-year cumulative rate of BCRL incidence was reported as 10% for SLNB+RT; 19% for ALND without RT; and 30% for ALND+RT [18]. Regardless of varying BCRL incidence rates, there are hundreds of thousands of women worldwide that struggle on a day-to-day basis with BCRL symptoms and millions who live in fear of developing it. Symptoms of BCRL vary in severity, with the greatest opportunity for successful management and prevention of progression in the subclinical stage [19]. 

### 1.3. Symptoms

Physical symptoms include swelling, leathery skin changes, heaviness, numbness, stiffness, and pain interfering with daily activities and negatively impacting functional well-being [20]. Symptoms present prior to the first occurrence of lymphedema have been most commonly reported as a feeling of tightness (e.g., jewelry or clothing too tight) and heaviness of the extremity [21]. Altered body image and feeling a loss of one’s previous self before breast cancer are major factors in causing psychological symptoms such as anxiety, depression, low self-esteem, and emotional distress [20,22]. Symptom distress and the burden of symptom management have been reported as factors in decreased quality of life for breast cancer survivors with BCRL [21,23].

The International Society of Lymphology [24] has classified lymphedema into four stages: 0–3, with no swelling represented by Stage 0; and lymphedema signs and symptoms with the most severity represented by Stage 3 (Table 1). Lymphedema severity (mild to severe) is classified by limb volume change based on contralateral limb comparison in unilateral cases. 

**Table 1:** Clinical staging and severity according to the International Society of Lymphology Consensus Document. Adapted with permission from International Society of Lymphology Executive Committee [24].

Clinical Stage	Description
0	A subclinical stage where swelling is not seen despite underlying changes in the lymphatic system
I	The initial stage of swelling which can be transient and where simple elevation can alleviate swelling
II	Swelling is constant and pitting without resolution using elevation
III	The tissue has become hard and fibrotic with associated skin changes
Severity	Based on volume differences between affected and contralateral limb in unilateral presentation
	Mild ≤ 20% increase; Moderate = 20%–38% increase, Severe ≥ 38% increase

Once a diagnosis of lymphedema is made, it requires a lifetime of treatment. There is no cure for lymphedema and an integrated approach across disciplines is needed for early lymphedema management, including early detection, education on risk-reduction and self-management, psychological and psychosocial support [25,26]. The inclusion of all individuals, practices, and centers that care for patients with breast cancer is imperative in order to provide the needed components of care for the best patient outcomes in managing breast cancer treatment, risk-reduction measures, and early detection of lymphedema [14,27,28].

## 2. Assessment and Management of LE

### 2.1. Assessment

There is a lack of consensus for standardized protocols in regard to measurement techniques. However, circumferential arm measurements, perometry, and water displacement are commonly used to detect and monitor arm swelling, although water displacement method is used less often due to its innate limitations and perometry is becoming more frequently used in breast centers [29,30]. Arm circumference measurements using a non-stretch tape measure is the most common means to measure limb girth (and volume through formula calculation) to monitor for change over time [31]. Both arms are measured at the hand and from the wrist to the axilla at specified anatomical landmarks or segments. The most common criteria for LE diagnosis is a change of 2 cm. difference between the affected and unaffected extremity at one anatomical point [29,32]. Perometry uses infrared light and optoelectronic sensors to assess limb volume and shape and records data for comparison over time [29,32]. Lymph volume differences of 10% or 200 mL between pre-op baseline and/or contralateral limb is the common criteria used in the diagnosis of LE [29,33]. Water displacement method offers sensitive and accurate volume measures and is able to accommodate for varying limb shapes due to swelling; however, it is contraindicated in patients with open skin lesions and hygienic precautions are not practical in many clinical settings [29,30]. More recently, bioelectrical impedance spectroscopy (BIS) and tissue dielectric constants (TDC) have been used as assessment tools for LE. BIS estimates extracelluar fluid volume by measuring resistance of body tissues to low alternating electric currents over various frequencies [32,34]. Resistivity coefficients for intra- and extracellular water of arms (*n* = 66) were used to predict water volumes using BIS in women with lymphedema (*n* = 23) and women without lymphedema (*n* = 13). BIS measures were highly correlated (*r* = 0.80–0.90) with total arm perometry measures and deemed an appropriate measure to detect or monitor BCRL [34]. TDC uses a probe connected to a control unit which displays tissue water changes when the probe is placed on the skin. Mayrovitz reported averaged TDC measures of the volar surface of the forearms were comparable to a single TDC measure, showing differences of +1 unit at a 95% CI [35].

Additionally, patient symptom report should always accompany objective measures and can be invaluable in detecting subclinical LE. Heaviness, swelling [21], and tingling and numbness [11] have been reported as the most common signs of impending development of LE. Once lymphedema has been diagnosed, it is important to rule out tumor recurrence or abnormalities with the venous system. This is usually done using lymphangioscintigraphy (LAS) prior to treatment [12].

### 2.2. Treatment 

There are no universally-applied, evidence-based treatment protocols to guide the treatment of BCRL [28,31,32,36]. Complete decongestive therapy (CDT), performed by specialty-trained lymphedema therapists, is currently considered the international standard of care for BCRL [3,14,24,28]. Patients are triaged to receive a combination of components that make up the CDT regimen. With the goal of therapy to move lymphatic fluid to an area where it can drain and reduce swelling, these components may include: manual lymphatic drainage (MLD), compression bandaging of the extremity, compression garments, exercise, and meticulous skin care [24,28,31,37,38]. Manual lymphatic drainage is a hands-on light, lymphatic massage that stimulates superficial lymphatic vessels to move lymph fluid from the extremity to an area where the lymphatics can drain properly [3,6]. 

#### 2.2.1. Non-Invasive Components of Care

Compression bandaging includes several layers of short-stretch bandages that cover the entire limb and create an effective gradient pressure to move lymph fluid out of congested areas [3,6,28]. Compression garments are personal garments that are properly fit by a trained specialist and worn on the affected extremity to maintain or prevent progression of swelling. These garments are worn long-term. Some women with BCRL have a garment for day wear and one with a stronger compression gradient to wear during sleep [3,28]. Exercise is prescribed depending on the severity of BCRL symptoms. Remedial exercises are prescribed initially when the goal is to reduce swelling in the extremity. Aerobic, strengthening, and flexibility exercises are prescribed in the self-management phase [3,28]. Skin care is essential for lymphedema management and includes meticulous hygiene and ongoing observation for breaks or texture changes in the skin [3,28,31,32]. It is imperative that providers educate patients about the signs and symptoms of infection (cellulitis) in order to identify and treat it at the earliest onset. Risk of infection is greater in patients with lymphedema due to the propensity of microbial growth in an environment of excessive protein-rich lymph fluid and dysfunctional lymphatics [32,39,40]. In the majority of patients, there is no antecedent event identified; therefore, knowledge of flu-like symptoms and the classic signs of acute inflammation are essential to facilitate early diagnosis of infection (cellulitis) and prompt treatment to prevent further impairment of lymphatic transport. Reducing the risk of infection and cellulitis can be enhanced by following the National Lymphedema Network risk-reduction guidelines [28,40]. Education about risk-reduction measures to avoid exacerbation of BCRL should also be included in patient teaching [28,31]. The first initial phase of intensive treatment under the supervision of a specialty-trained therapist is followed by a transitional phase, in which patients learn to manage and implement their own treatment, using individual or bundled components. Promotion of adherence to long-term control with self-management regimens is critical. 

#### 2.2.2. Surgical Treatment

Advances in surgical procedures are currently under investigation for both prevention and treatment of lymphedema using excisional operations, lymphatic reconstruction, and tissue transfer [2,31,32,41]; however, surgery is not considered first-line treatment for LE. Patients who undergo surgery must be counseled on the surgical risks and understand that lifelong compression garments are usually prescribed post-operatively and as maintenance [2,31,41]. Currently, surgical treatment with excisional operations, such as debulking procedures and liposuction, has been used with success to alleviate symptoms from severe lymphedema. Experimental work in creating lymphaticovenular anastomosis (LVA) to bypass lymphatic obstruction is the newest in surgical advances with supramicrosurgical lymphatic reconstruction. Tissue transfer procedures involving lympho-lymphatic anastomosis and lymphatic grafting and use of other types of tissue such as omentum, muscle, or skin flaps have been used to channel lymphatic flow [2,5,31,41]; however, Vignes* et al.* reported 15 complications in 10 patients who received autologous lymph-node transplantation (ALNT), warranting more studies to determine the indications and contraindications for ALNT [42]. Cormier* et al*. performed a systematic review of 20 surgical studies and reported that, while promising, no studies show a clear benefit of surgery over CDT for the treatment of BCRL [43]. Damstra* et al.* also reported the lack of standardization of research in a systematic review of the literature in regard to LVA [44]. Granzow* et al.* recently reported a literature review over the past ten years indicating that treatments have evolved to become more effective and less invasive [45]; however, future studies are needed with larger sample sizes in order to determine best surgical practices in the prevention and treatment of BCRL. 

#### 2.2.3. Nodal Status Determination

Breast cancer surgery has historically included nodal biopsy and removal in order to detect metastasis and aid in staging. With the advent of sentinel lymph node biopsy (SLNB), studies have changed surgical protocols in regard to the necessity of axillary lymph node dissection (ALND) and in decreasing the number of nodes necessary for ALND. The American College of Surgeons Oncology Group (ACOSOG) Z0011 trial (*n* = 891) reported no benefit to ALND in women who underwent lumpectomy and whole breast irradiation and had T1-T2 invasive breast cancer, 1–2 positive sentinel lymph nodes, and no palpable lymphadenopathy [46]. There is also growing evidence using diagnostics; for example, axillary reverse mapping (ARM) which helps surgeons spare arm-draining lymph nodes when performing sentinel lymph node biopsies [8]. Most recently, Douek* et al.* reported findings from the SentiMAG Multi-center Trial (*n* = 160) that demonstrated the feasibility of using superparamagnetic iron oxide contrast agent injected subcutaneously into the breast and detection of the sentinel node with a magnetometer. The technique was not inferior to the traditional SLNB procedure with an identification rate of 94.4% to 95% respectively. Contraindications for inclusion in the trial were patients with a hypersensitivity to iron or dextran compounds, magnetic tracers, or superparamagnetic iron oxide contrast agents, iron-overload disease, and patients with implantable devices or pacemakers on the chest wall [47].

## 3. Adjunct Therapies

### 3.1. Pneumatic Compression Therapy

In addition to CDT as front-line treatment of BCRL, adjunct therapies are used for symptom management and risk reduction. These include the use of pneumatics, aqua lymphatic therapy (ALT), complementary and alternative medicine (CAM), exercise, and low-level-laser therapy (LLLT). Intermittent pneumatic compression (IPC), consisting of a sleeve garment with chambers that apply pressure, simulating the work-and-release method of manual compression, is used in reducing edema [36]. Ridner* et al.* studied the use of pneumatic compression in comparison with self-administered manual lymphatic drainage for treatment of lymphedema. Although there were no statistically significant differences between the groups (*n* = 155), 95% of patients reported a self-perceived positive limb volume outcome with IPC in the home setting [48]. In a pilot study to evaluate lymphatic response to pneumatic compression therapy in normal control and BCRL subjects (*N* = 9; control: 3 and BCRL subjects: 6) using near infrared (NIR) fluorescence imaging, Adams* et al*. reported greater lymphatic propulsion in 4 of 6 BCRL-affected arms after the use of IPC with a segmented sleeve with a calibrated gradient processor. NIR fluorescence imaging was used to measure lymphatic propulsion, velocity, and lymphatic vessel recruitment before, during, and after pneumatic compression therapy. The piloted use of NIR fluorescence imaging in this study allows for the use of microdosing, which decreases the potential for adverse reactions. Adams* et al*. also discussed the use of this image technology as brighter or more easily visualized and having improved sensitivity, as compared to planar NIR devices [49]. 

### 3.2. Aqua Lymphatic Therapy 

Aqua lymphatic therapy is a method that uses the viscosity of water to provide resistance to body movement. Hydrostatic pressure is used to protect the arm from swelling and reduces edema. Groups of patients with BCRL attended 45-min sessions in a pool and performed breathing and self-massage techniques in the water in sequence. An immediate mean arm volume reduction of 16% (53 mL) of the affected arm was reported after the first ALT session, with a reduction of 29% (98.2 mL) after the last ALT session (*n* = 16 ALT; *n* = 32 Control) [50]. 

### 3.3. Complementary and Alternative Medicine 

Complementary and alternative medicine (CAM) is used by as many as 75% of women with breast cancer as a means to cope with side effects from conventional treatment, find solace, and facilitate healing and cure [51]. Wanchai, Armer, and Stewart performed a systematic review of CAM use by women with breast cancer. Thirty-three articles identified natural products (e.g., herbs, minerals, vitamins, food) and mind/body medicine (e.g., meditation, prayer, humor) as the most frequently used types of CAM by women with breast cancer. Most of the studies were conducted in the US (52%), but included Canada (15%), China (9%), and Australia (6%) [51]. Body-based CAM (e.g., massage, energy work, acupuncture) is gaining more popularity, but can have adverse side effects. Massage therapy should not be applied to truncal borders and deep tissue massage is contraindicated for patients who are at risk for the development of BCRL due to the possibility of damaging the lymphatic structures [51]. Wanchai* et al.* reported that communication and referral to CAM resources were found to be mainly through family and friends, rather than health care providers [51]. 

### 3.4. Low-Level-Laser Therapy 

Low-level-laser therapy (LLLT) is another treatment modality that has been studied as an adjunct or alternative in reducing fluid volume and improving arm function in women who have BCRL; however, there are limitations to these studies in regard to sample sizes and differences in measuring objective outcomes [52]. In a systematic review of 41 studies of which only 4 studies met eligibility and evidence point criteria, Lima* et al*. reported that all studies showed favorable results in limb volume reduction; however, there were many methodological limitations [53]. More recently, Ridner* et al*. reported results of a pilot three-group RCT (*n* = 46) in which LLLT with compression bandaging demonstrated equal volume reduction in half the time as MLD. Advanced practice nurses were trained to administer the treatment regimens in this pilot study which demonstrated that APNs could effectively treat lymphedema, decreasing time and burden on both the patient and therapists [54]. Ridner* et al*. discuss the need to compare lymphedema therapy given by lymphedema-trained therapists, RNs, and occupational therapists with and without certification [54]. It would seem feasible to train additional health care providers to deliver lymphedema therapy to accommodate the large number patients with LE; however, there is currently no fee schedule for billing these services by healthcare providers (such as nurses or massage therapists) who are not covered under physiotherapy billing codes. 

Regardless of the type of adjunct therapy, it is important patients communicate with health care providers to prevent possible adverse effects from combining therapies, prevent injury, and discuss resources that can most benefit patients.

## 4. Predisposing Factors

There is more consistency needed in defining BCRL risk factors; however, the following are currently included in the literature including: (a) obesity (BMI ≥ 30; (b) sedentary lifestyle; (c) breast cancer surgery; (d) radiation therapy (XRT); (e) post-surgical infection; (f) radiation skin reaction; (g) age; (h) comorbid conditions and medication usage; and (i) genetic predisposition [28,31,55]. Obesity has been reported as a risk factor for BCRL; however, it is a modifiable risk factor with diet and exercise [56]. Ridner* et al.* reported an approximate 3.6 times likelihood of developing BCRL at 6 months or greater after breast cancer diagnosis in women whose BMI was ≥30 than patients with a BMI < 30 [57]. 

### 4.1. Exercise

There are many precautions in preventing BCRL that potentially lead to a sedentary lifestyle due to fear of developing lymphedema with strenuous activity. A systematic review of 19 studies by Kwan* et al.* [58], of which seven studies addressed resistance exercise, seven addressed aerobic and resistance exercise, and five addressed other exercise modalities, reported findings that supported the safety of resistance exercise without an association with the development or exacerbation of BCRL [58]. Schmitz* et al.* conducted a RCT of progressive weight lifting in women with BCRL (*n* = 141) [59]. Women participated in a supervised program twice weekly over one year. Findings reported participants in the weight-lifting group demonstrated increased strength and there was a decrease in lymphedema exacerbations and symptoms [59]. 

### 4.2. Breast Cancer Surgery and Radiation Therapy

Breast cancer surgery, ALND, SLNB, and radiation therapy are the main risk factors for the development of BCRL due to trauma to the lymphatic structures. Less invasive surgical procedures and the use of SLNB has helped to decrease the incidence of BCRL; however, there are still significant risks of developing BCRL with SLNB alone or no axillary surgery at all [4,12,55]. 

### 4.3. Comorbidities

Cancer survivors are living longer, with an estimated 13,027,914 people currently living among all reported cancer sites in the United States. Based on 2004–2010 SEER data, 89.2% of breast cancer survivors are estimated to live at least 5 years [60]. Age is associated with decreased function and functional independence [61]. Bellury* et al.* found an interaction between symptom burden and comorbidities in 39% of older breast cancer survivors studied (age ≥ 70 [*n* = 759]), and support a gero-oncology survivorship paradigm to guide care-planning and delivery [61]. Comorbidities associated with breast cancer survivors with BCRL (*n* = 74) compared with breast cancer survivors without BCRL (*n* = 75) were studied by Ridner and Dietrich [62]. Findings identified obesity, orthopedic problems, hypertension, and arthritis as more prevalent in the lymphedema group [62]. Although there are limitations in determining causality due to pre-existing conditions in some of the participants, the findings suggested that being sedentary, compromised cardiovascular status, and inflammatory processes in the development, or consequences, of lymphedema warranted further investigation [62]. Co-association of medications ordered to manage these conditions may also be a factor in lymphedema emergence, progression, and management [63].

### 4.4. Genetic Predisposition

In addition to age, genetic predisposition is a non-modifiable risk factor that has gained increased attention. If patients can be identified as high risk for the development of BCRL from a genetic standpoint, it will help establish specialized monitoring and fast-track clinical management. Newman* et al.* identified two genetic loci and a third gene that possibly confer genetic predisposition to secondary lymphedema [64]. More studies are necessary to determine genetic associations that will lead to more effective prevention and management strategies. 

## 5. Risk-Reduction Strategies and Preventive Interventions

Physical means by which women with BCRL can perform risk-reduction measures include: (a) protection of skin (e.g., keep skin clean and dry; avoid punctures such as blood draws, injections, or intravenous infusions; observe for signs and symptoms of infection; and wear gloves when gardening); (b) exercise; (c) avoid extreme temperatures; (d) avoid limb constriction; and (e) wear well-fitted compression garments when prescribed [31,32]. Self-management is a responsibility of patients, which usually involves performing one or more interventions to control or prevent symptoms. 

### 5.1. Self-Management

The ability to care for oneself by self-administration of a prescribed regimen to regulate symptoms and promote well-being encompasses self-management [22]. Self-CDT or MLD is often performed by women with BCRL to facilitate lymph circulation of the affected extremity. A category of CAM, mind/body interventions, is used to promote relaxation and minimize stress. Although there is little to no evidence on the impact of mind/body interventions on outcomes measures for BCRL, there has been increasing interest in more rigorous study in this area. Some of these interventions include tai chi, yoga, meditation, mindful-based stress reduction (MBSR), acupuncture or acupressure, and exercise. A quasi-experimental, pre- and post-test control group design was used to study MBSR with breast cancer survivors (*n* = 32) [65]. The participants in the intervention group took an 8-week MBSR training course which, when compared to the control group, demonstrated a statistically significant decrease in systolic and diastolic blood pressure immediately following MBSR and at 1-month follow-up, although it did not remain statistically significant at completion. A state of mindfulness was statistically significant throughout the study. Morning and afternoon cortisol levels were also measured with participants and, although not statistically significant in comparison at the completion of the study, a clinical significance was noted with the intervention group’s self-report of decreased stress [65]. The stress of breast cancer is compounded by a diagnosis of BCRL and can often contribute to failure to perform self-care; therefore, MSBR may be helpful in reducing risk and improving management of BCRL [66]. As with most of the CAM studies reviewed, RCT with larger sample sizes are recommended to further evaluate effectiveness in treating and preventing BCRL. 

#### Exercise and Weight-Lifting 

Exercise and weight-lifting have been reported as beneficial in women with BCRL, provided that training and conditioning are supervised. The Physical Activity and Lymphedema (PAL) Trial was a pivotal point for women with BCRL who were fearful of physical activity. The PAL Trial reported that progressive weight-lifting demonstrated a decrease in exacerbation incidence, reduced symptoms, and increased strength with no significant effect on limb swelling [59]. In addition, evidence suggests that resistance exercise is safe without an increase in BCRL risk for breast cancer patients [58]. In a prospective breast cancer morbidity trial, arm volume measures were performed preoperatively and at three month intervals post-surgery on 196 women of whom 43 developed lymphedema. For an upper arm volume increase ≥3%, women were prescribed a compression garment to wear daily for 4 weeks. Findings reported that the use of a compression garment was effective in treating subclinical arm volume (48 mL decrease; *p* < 0.0001) in a single-arm pilot [19]; however, additional research studies with larger sample sizes and longer follow-up are necessary to establish compression garments as an evidence-based method of reducing BCRL risk. 

### 5.2. Surgical Prevention 

Surgical means for prevention of BCRL has been reported using the lymphatic microsurgical preventive healing approach (LYMPHA) technique during node dissection [67]. The LYMPHA approach is a technique by which the lymphatics are temporarily clipped in preparation for microsurgical anastomosis to one or two collateral branches of the axillary vein, without damage to lymphatic pathways [67]. In a prospective study, Boccardo* et al.* randomized 46 women into two groups: one receiving the LYMPHA technique and a control group who had no preventive surgical approach. The patients underwent lymphoscintigraphy pre-operatively and at 18 months post-operatively and were followed with volumetry post-operatively at 1, 3, 6, 12, and 18 months. Lymphedema appeared in one patient in the treatment group at 6 months follow-up compared to seven patients in the control group. Arm volumes in the control group demonstrated a significant increase at every follow-up time point, while no statistically significant changes in volume were assessed in the treatment group [67]. Reproducible results using the LYMPHA approach with larger sample sizes could change traditional approaches to node dissection, preventing secondary lymphedema in breast cancer patients.

## 6. Psychological and Psychosocial Symptoms

Psychological and psychosocial symptoms contribute to poor adherence with self-management regimens to control BCRL symptoms [14,20]. Symptom distress results from the disruption in daily life and women with BCRL are often faced with multiple symptoms that require complex self-management regimens [11,23]. The burden of BCRL symptoms and self-management often elicit symptoms of anxiety, depression, emotional distress, diminished sexuality, and feelings of sadness [11,23]. Rosedale and Fu reported a secondary analysis of phenomenologic data with breast cancer survivors (*n* = 13) in which temporal, attributive, and situational dimensions of symptom distress were identified [68]. They report lingering symptoms that were expected to disappear; unexpected identity issues in which the post-cancer self was viewed as inferior to the pre-cancer self; and unexpected situations such as those precipitated by symptoms associated with BCRL development [68]. Fu* et al.* reported that factors associated with a negative psychosocial impact combined with psychological symptoms resulted in poorer social well-being in breast cancer survivors with BCRL [20]. Negative psychosocial impact was described by women with BCRL who had experienced unsupportive work environments, perceived social abandonment and social isolation, inability to participate in family leisure activities, marginalization, perceived diminished sexuality, public insensitivity, and financial burden [20,69,70]. The lack of instruments that can accurately measure subjective data relevant to the negative impact of psychological and psychosocial issues in women with BCRL is challenging; however, health care providers can be a positive influence by providing a supportive-educative element to care, promoting self-management behaviors, and staying cognizant of internal and external resources to help alleviate symptom distress [25,26]. 

### Impact on Patients and Families

Literature suggests that the diagnosis of chronic disease has an impact on individuals and their families [71]. Lymphedema has been reported to have an adverse effect on family functioning [71,72]. Factors that determine how a family responds and copes with stressors are dependent on: (a) the relationship between the family members, especially immediate members; (b) functionality and flexibility of the family unit; (c) availability of resources (e.g., friends, community); and (d) the number of stressors [71,72]. Radina and Armer performed interviews to determine issues of impact of lymphedema diagnosis in the context of family. They used the Family Adaptation and Adjustment Response model to interpret their findings as follows: (a) family adjustment to changes is a major factor with family functioning; (b) task modification with daily activities can be changed in the way they are done or someone else may be needed to help; (c) following risk-reduction guidelines is important to achieve an understanding of self-management; (d) identifying resources to help achieve effective coping strategies; and (e) maintaining family routines to prevent risk of family functioning imbalance [71]. In addition, Radina performed interviews with women breast cancer survivors with BCRL (*n* = 27) in the context of family leisure and reported modifications to activities and refraining from dangerous or difficult activities as patterns of coping [70].

## 7. Supportive Care

Several studies in the literature focus on patient-education; however, few incorporate a supportive component. Education is important for patients and health care providers alike; however, education alone is not sufficient to provide the support that patients need [22]. It is important to provide resources that address psychological and psychosocial needs, as well as the availability of internal and external resources (e.g., lymphedema therapists, support groups, medical equipment, printed information and/or other media). Increasing the awareness and education of breast cancer survivors early after diagnosis should be implemented about BCRL, risk factors for the development of BCRL, risk-reducing activities, and self-management, if necessary. Due to the increasing numbers of cancer survivors, supportive-educative programs will play a pivotal role in the quality of survivorship [73]. 

### 7.1. Adherence to Self-Management of BCRL

Self-care management can be complex and require education on several components. Motivational interviewing and interactive teaching strategies have been used to enhance the educational process. Armer* et al.* describes the use of motivational interviewing in helping breast cancer survivors become empowered in their own care [25]. Solution-focused therapy is also helpful in motivating patients and in identifying patient goals of self-management [25]. Patient engagement is a key factor in reducing risk and symptom management of BCRL. Participatory learning methods such as interactive theatre have been used to engage patients in discussion regarding breast cancer issues [74]. Further research is needed to develop reliable and valid instruments that will determine the effectiveness of these innovative approaches. 

### 7.2. Health Care Provider Knowledge

The importance of health care provider knowledge about BCRL is paramount to developing interdisciplinary programs of care. The numbers of patients who see cancer-related physicians are declining each year as patients move into the long-term survivorship phase of care [75]. Tam* et al.* reported results of a study of 887 oncologists, primary care physicians, and surgeons who took a web survey on knowledge of BCRL, education, and referral patterns [76]. Oncologists and surgeons scored highest in knowledge and education categories. Referrals were also highest in the oncologist and surgeon group, with only 36.2% of primary care physicians reported as ever having made a referral for BCRL. The response rate for this study was 35.9% and although referral information was not specific to time periods, the conclusions identified that educational programs, handouts, and emails were the preferred methods of staying current with BCRL information. With breast cancer survivors now living longer, oncologists and surgeons who typically follow patients long-term are beginning to feel comfortable with patients returning to their primary physicians for follow-up. This movement back to primary physicians is significant, especially if they are not informed about BCRL risks, treatment, and supportive care interventions [73,76]. The clinical implications of better-informed health care providers and patients in regard to managing BCRL are high priority because of the positive impact on patient outcomes; however, education does not stop with symptom management. There are cultural differences and similarities that need to be taken into account, due to the diversity of the population in the US; therefore, health care providers need to practice culturally-specific care [77]. Patients who feel that their cultural beliefs have been included as part of their plan of care tend to be more satisfied with care and are more apt to be compliant with treatment [78].

### 7.3. Palliative Care

Educative-supportive systems are also integral to providing palliative care. The World Health Organization (WHO) defines palliative care as that which integrates physical, psychological, and spiritual aspects of care with the goal of providing symptom relief and quality of life for both patients and their families [79]. In a systematic review of the literature related to the effectiveness of cancer-related lymphedema management in the palliative care setting, eleven articles were selected. Beck* et al.* reported all studies were rated in the category of “effectiveness not established” and reported the need for larger, well-designed studies; however, closed-controlled subcutaneous drainage was deemed as potentially effective in patients with late-stage cancer [80]. A CDT program may be beneficial; however, treatment should be carefully evaluated by a trained therapist. The pathophysiology of late-stage cancer and accompanying comorbidities require adaptation and sometimes elimination of treatment components [81].

## 8. Interdisciplinary Surveillance

An interdisciplinary approach to care and monitoring of patients who have been diagnosed with breast cancer is a standard of the American College of Surgeons’ National Accreditation Program for Breast Centers [82]. With over 200,000 cases of breast cancer cases estimated in 2014 by the American Cancer Society [83], and as many as 40% of the 2.5 million breast cancer survivors in the United States who will develop BCRL [7,32], it is important to incorporate all disciplines involved in the care of patients in plans of care that emphasize early detection of BCRL, other comorbidities, and/or recurrence of disease. A seamless plan of care initiated pre-operatively gives health care providers the opportunity to detect BCRL symptoms early, when there is a greater chance to prevent progression in the subclinical stage [19], and provides a continuous plan of care from the inpatient to outpatient settings. 

### 8.1. Measures for Surveillance

The most commonly used reliable and valid physical measures used to diagnose and monitor BCRL have been studied [12,29,30,31]; however, there is little research that incorporates subjective measures relevant to quality of life with objective measures to determine a relationship with early detection of BCRL. Two reliable and valid instruments, the Lymphedema Breast Cancer Questionnaire (LBCQ) [21,29,84] and the Functional Assessment of Cancer Therapy-Breast (FACT-B+4), have been used to monitor symptoms and evaluate health-related quality of life (HRQOL) [85,86]. There has been development of several valid and reliable tools to measure QOL issues, such as the 22-item Functional Living Index-Cancer (FLI-C) [87]. The LYMQOL is another QOL measure specifically developed for patients with limb lymphedema that has proven to be useful [88].

#### 8.1.1. Lymphedema Breast Cancer Questionnaire (LBCQ) 

The LBCQ is a 58-item semi-structured questionnaire designed to assess indicators of lymphedema, their frequency, and symptom management strategies [12,21,29,84]. The LBCQ has established reliability and validity using Kuder-Richardson-20 and the test-retest method, showing an acceptable measure of internal consistency (*r* = 0.785). Test-retest reliability was reported high (*r* = 0.98) using healthy women (*n* = 35) with a 2-h test-retest interval and an acceptable internal consistency [21]. 

#### 8.1.2. Functional Assessment of Cancer Therapy (FACT-B+4)

The FACT-B is a 44-item self-report instrument for measuring multi-dimensional quality of life with patients who have breast cancer. It incorporates physical, functional, and emotional well-being. Brady* et al.* demonstrated test-retest reliability and validity of the FACT-B with two validation samples (alpha = 0.90 with subscale alpha coefficients ranging from 0.63 to 0.86) [85]. Coster, Poole, and Fallowfield designed and tested a supplement to the FACT-B, a 4-item arm subscale to assess the impact of arm morbidity following breast cancer surgery [86]. The FACT-B+4 was validated on clinical trial participants (*n* = 279 women in a trial of sentinel node guided axillary therapy and *n* = 29 women attending a lymphedema clinic). Internal consistency (alpha coefficient = 0.62 to 0.88) and test-retest reliability (*r* = 0.97) were high. The sensitivity of the instrument over time was validated with a subset of 66 participants who completed three assessments [86].

### 8.2. Innovation in Qualitative Measures

An online version of the LBCQ and FACT-B+4 is an innovative application that has been developed for Ellis Fischel Cancer Center (EFCC) at the University of Missouri-Columbia, sponsored by the American Lymphedema Framework Project (ALFP) website. A prospective surveillance study using online LBCQ and FACT-B+4 instruments in conjunction with objective measures has been initiated with an aim to demonstrate earlier detection of BCRL and improved QOL and functional outcomes compared to standard care (Table 2). 

## 9. Clinical Research

Breast cancer-related lymphedema incidence will continue to rise, despite less invasive surgical methods and the use of SLNB. Cancer survivors are living longer; increasing numbers of functional limitations and comorbidities, such as immobility and obesity, further increase the risk for the development of BCRL. There are no evidence-based methods of measurement and treatment, or consensus on BCRL guidelines for palliative care. There is a need for better methods for early detection of subclinical BCRL, as well as improvement with adherence to self-management regimens. Currently, there are at least three clinical trials in progress administered by NCI-funded national oncology clinical trials (Table 3). There is a scarcity of rigorously-conducted comparative research studies related to patients with breast cancer-related lymphedema, limiting the development of evidence-based assessment and treatment for hundreds of thousands of women who have or are at risk for the development of BCRL.

**Table 2:** Ellis Fischel Cancer Center Study Model: Early detection of BCRL with early intervention in the clinical setting.

Aims	(1) To determine if rigorous surveillance with web-based questionnaires in conjunction with arm volume measurements demonstrates earlier detection of BCRL compared to standard care. (2) To determine if earlier detection of BCRL using web-based HRQOL questionnaires in conjunction with arm volume measures improves QOL and functional outcomes. (3) To assess the cost and efficiency of nursing time in completing and evaluating questionnaires
Methods	Longitudinal, prospective, mixed-method study which will recruit women with newly-diagnosed breast cancer to complete a comprehensive symptom and quality of life assessment (LBCQ) and undergo arm volume measures pre-operatively, post-operatively, and quarterly. Preoperatively and at 12-month follow-up, patients will complete the FACT-B+4 questionnaire. Descriptive statistics and correlations will be used to examine relationships between actual measured arm volumes and subjective web-based questionnaire responses to determine if early symptoms correlate with limb volume changes. This study has been approved by the University of Missouri Health Sciences Institutional Review Board.
Results	Currently recruiting (n = 25 currently have given informed, written consent; up to 100 will be recruited)
Conclusion	Early diagnosis and intervention for BCRL is reported to improve the patients’ quality of life and functional outcomes. By utilizing a novel electronic tool developed in our institution (LBCQ) as a component of rigorous surveillance, we hope to show lymphedema is diagnosed earlier than with standard care, resulting in improved HRQOL and functional outcomes for our patients.

**Table 3:** Randomized clinical trials for lymphedema.

Clinical Trial Organization	Clinical Trial Number	Name of Trial	Principal Investigator(s)
Alliance (CALGB)	70305	A randomized education/exercise intervention study to reduce risk of lymphedema in women treated for breast cancer	Electra Paskett, Jane Armer, Lisa Yee, Michele Naughton,* et al.*
Alliance (ACOSOG)	Z1070 Successor Trial	Axillary management of T0-T3 node positive breast cancer receiving neoadjuvant chemotherapy	Judy C. Boughey MD, Tom Buchholz MD, Bruce Haffty MD, Vera Suman PhD, Janice Cormier, MD, MPH, Jane Armer, RN, PhD
Gynecologic Oncology Group (GOG)		The Lymphedema and Gynecologic Cancer (LEG) Study: Incidence, Risk Factors, and Impact	Richard Barakat, MD, MSKCC, NCI R01

## 10. Conclusions

A multidisciplinary approach to surveillance of BCRL should be instituted as a means of secondary prevention with all women diagnosed with breast cancer. A pre-operative history and physical, including bilateral arm volume measurements, allows for baseline information that is reliable as a prognostic indicator with post-operative and future interval measurements. A baseline pre-operative measurement is more reliable because there is no surgical swelling to inhibit accuracy [28,29]. Pre- and post-operative limb volume measurements at regular intervals, such as quarterly for 12 months, semi-annually for 1–3 years, and then annually, thereafter, could identify LE at its earliest state, when there is a better chance for prevention of progression [19,31]. In addition, education about predisposing factors for BCRL, risk-reduction activities, and resource availability should be made available pre-operatively [89]. Birkballe* et al.* conducted a clinical perspective analysis at a multidisciplinary lymphedema center at the Copenhagen Wound Healing Centre [90]. Data were collected for the first 4–5 years after the center was established on 8,058 patient consultations. Birkballe* et al.* reported 31% of patients had never had any diagnostic testing or treatment for lymphedema prior to referral [90]. In addition, 86% of the patients required multidisciplinary assessment [90]. The center is staffed by two nurses who specialize in lymphedema with required training (research and clinical), two physicians one day a week (one resident with an interest in lymphedema and a dermatologist), and dedicated clinical physiologists for diagnostics and imaging services. Birkballe* et al.* reported that an organized and planned utilization of a multidisciplinary staff at their center improved management, knowledge, and awareness of lymphedema [90]. This model is an example of a successful model that, with minor changes, could be adapted to patients in the US as a supportive-educative program for surveillance and ongoing support of self-management for controlling symptoms of BCRL. 

### 10.1. Prospective Surveillance Model

International efforts have contributed to bringing lymphedema to the forefront of research and recognition. Multidisciplinary best practice templates and guidelines based on expert opinions have been developed in a number of organizations, such as the International Society of Lymphology (ISL) [24], International Lymphoedema Framework (ILF) [38], European Wound Management Association (EWMA) [91], American Lymphedema Framework Project (ALFP) (e.g., [3,12,31,36,43,58,80]), and others. A prospective surveillance program for BCRL (PROSURV-BCRL) is proposed in Table 4. It is based on available recommendations by the above-mentioned International organizations, as well as the National Lymphedema Network [28], Best Practice Guidelines [31], Stout* et al.* [19,27], Schmitz* et al.* [59], Birkballe* et al.* [90], and many experts in the field. Larger studies are needed to determine a greater advantage of the proposed program over the medical model currently used. The components are not necessarily different, except in terms of the time frame in which the program is initiated. 

### 10.2. Costs Associated with Traditional and Prospective Models

Traditionally, medical models have been used that addressed lymphedema symptoms when patients reported them at provider follow-up visits or when visible swelling, pain, or decreased function caused the need for a problem-oriented visit. Stout* et al.* provided a detailed estimation of direct treatment costs of BCRL treatment with 100 women over a period of a year using extrapolated clinical scenarios [27]. The costs were compared between a prospective surveillance model and a traditional model. A sensitivity analysis comparing varied incidence rates between the two models was also performed, as well as an analysis of the effect that BCRL progression might have on their assumptions. Although direct cost data are lacking and further analysis is necessary, Stout* et al.* reported a potential decrease in direct costs of a prospective surveillance model, compared to a traditional model related to management of BCRL [27]. Upfront costs of a prospective surveillance program and dedicated staffing would be significant and may be a deterrent; however, forward thinking past short-term gains and commitment to early detection and prevention of BCRL is predicted to result in significant long-term savings from a financial standpoint. Most importantly, patients may experience a higher quality of life.

**Table 4:** PROSURV-BCRL Model (Visits coordinated with MD appointments).

Pre-op assessment with every woman who will undergo breast cancer surgery and/or RT.	H&P; height, weight, BMI, baseline bilateral limb (arm) volume measures with certified lymphedema therapist, functional assessment; provision of educational and resource information (predisposing factors, risk-reduction activities, signs and symptoms of early BCRL, support group information, team contact information; physical activity assessment and program information; nutritional information); introduction to team members and Q & A opportunities
Post-operative visit by team nurse and lymphedema therapist	Bilateral LV measure and assessment; supportive-educative visit; Ongoing: assess eligibility for clinical trials
Interval visits @ 1, 3, 6, 9, 12 months	Weight; BMI; bilateral LV measures; functional assessment; physical activity assessment; skin assessment. Ongoing: physical exercise program options, unless contraindicated; weight management program options; nutritional support and referral with dietician; support group information; virtual support group/blogs; contact with patient by team member monthly the first year post-breast cancer treatment; psychological/psychosocial assessment/Family assessment with a trained counselor.
Semi-Annual visits for 1–3 years	Same as above with team contact every 2 months to discuss status and evaluate need for resource referral.
Annual visits, if no BCRL is diagnosed	Team contact quarterly to discuss status and evaluate need for resource referral.
If BCRL is diagnosed:	Imaging: (lymphoscintigraphy/lymphangioscintigraphy, lymphography, MRI, ultrasound) to assess and/or rule out problems with lymphatic structures and flow and venous circulation, if indicated. Ongoing: Visits per treatment plan prescribed by lymphedema therapist for initial CDT.
	Ongoing: support of self-management: Team contact every month or more often initially if necessary, treatment information, clinical trial information if available, support group information/other media information, new product information, Online LBCQ q 3 months or per institutional protocol, Fact-B+4 annually or per institutional protocol
Ongoing throughout survivorship:	Celebration events at least annually

### 10.3. Future Research

A commitment to high-level clinical research is needed to test treatment protocols in order to develop evidence-based guidelines. The achievement of standardization is a step that has to be taken in order to move forward in developing clinical trials with larger numbers of participants. Improved patient outcomes can only be quantified with valid and reliable measures. Minimum data sets (MDS) are currently under development; these will be used by researchers as a repository for de-identified research data that will be available for outcome comparisons on national and international levels. Chi-Ren Shyu, Director of the University of Missouri Informatics Institute, and recipient of National Library of Medicine funding, has designed an internet-based system to collect, collate, transfer, and analyze data [31,92]. Collecting, organizing, and analyzing data with findings that can be disseminated worldwide is a major achievement in our priority to facilitate global health. The increase in higher-level studies will enable us to shift the paradigm of care for breast cancer survivors with BCRL to a more patient-centric and effective preventive approach. 
